# A case of an advanced renal collecting duct carcinoma in which initial therapeutic effect was achieved with pembrolizumab plus axitinib

**DOI:** 10.1002/iju5.12666

**Published:** 2023-11-08

**Authors:** Yuichiro Atagi, Kei Daizumoto, Shinsuke Mohri, Kai Somiya, Daisuke Seto, Shigeo Nakanishi, Yutaka Yanagihara, Iku Ninomiya, Kenjiro Okamoto, Sadamu Yamashi

**Affiliations:** ^1^ Ehime Prefectural Central Hospital Matsuyama Ehime Japan; ^2^ University of Tokushima Graduate School of Biomedical Sciences Department of Urology Tokushima City Tokushima Japan

**Keywords:** axitinib, cabozantinib, immune checkpoint inhibitors, pembrolizumab, renal collecting duct carcinoma

## Abstract

**Introduction:**

Renal collecting duct carcinoma is often found in advanced cancers and has a poor prognosis. Here, we present the case of symptomatic metastatic collecting duct carcinoma in which we observed an initial therapeutic effect of immune checkpoint inhibitors plus tyrosine kinase inhibitors.

**Case presentation:**

The patient was a 69‐year‐old male who was referred to our hospital for examination of a right chest tumor and related pain. Contrast‐enhanced computed tomography and tumor biopsy were performed, leading to a diagnosis of collecting duct carcinoma. A combination of pembrolizumab plus axitinib was initiated as first‐line therapy; right chest pain decreased, and tumor shrinkage was observed. Seven months after treatment initiation, tumor progression was noted. Cabozantinib was initiated as second‐line therapy; however, was discontinued due to patient fatigue. The patient died 15 months after the initiation of treatment.

**Conclusion:**

For symptomatic metastatic collecting duct carcinoma, pembrolizumab plus axitinib may have initial therapeutic effects.

**Abbreviations & Acronyms**
CDC: (renal) collecting duct carcinoma
CK20: cytokeratin 20
CK7: cytokeratin 7
CT: computed tomography
E‐cadherin: epithelial cadherin
EMA: endomysial antibodies
ICI: immune checkpoint inhibitors
irAEs: immune‐related adverse effects
PD: progressive disease
PD‐L1: programmed death‐ligand 1
PFS: progression‐free survival
PR: partial response

**Keynote message**

Treatment of pembrolizumab plus axitinib was effective for a patient with advanced renal collecting duct carcinoma.

## Introduction

CDC is a rare tumor among renal cell carcinomas. Metastatic CDC has a poor prognosis, and no standard treatment has been established. We report a case of metastatic CDC in which an initial therapeutic effect was achieved using pembrolizumab plus axitinib.

## Case presentation

**Fig. 1:**
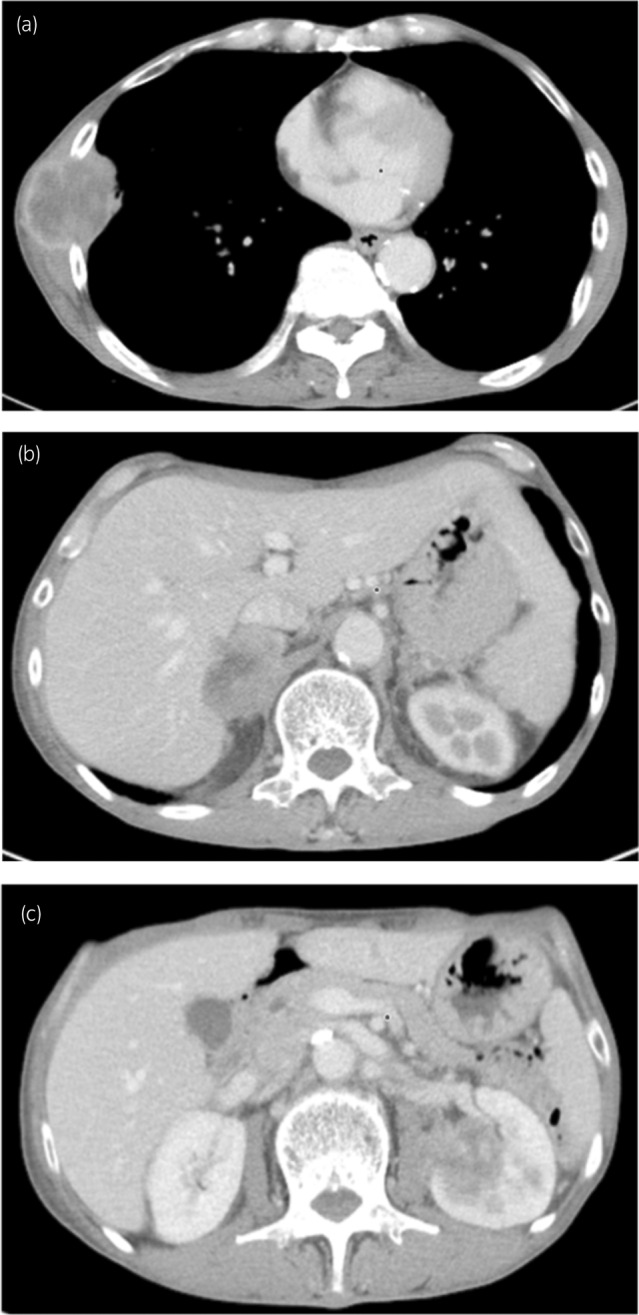
CT image on admission. (a) A 4 × 4 cm mass is visible on the right thoracic wall. (b) A mass is seen on both adrenal glands. (c) A tumor with contrast enhancement and irregular margins is visible on the left kidney.

**Fig. 2:**
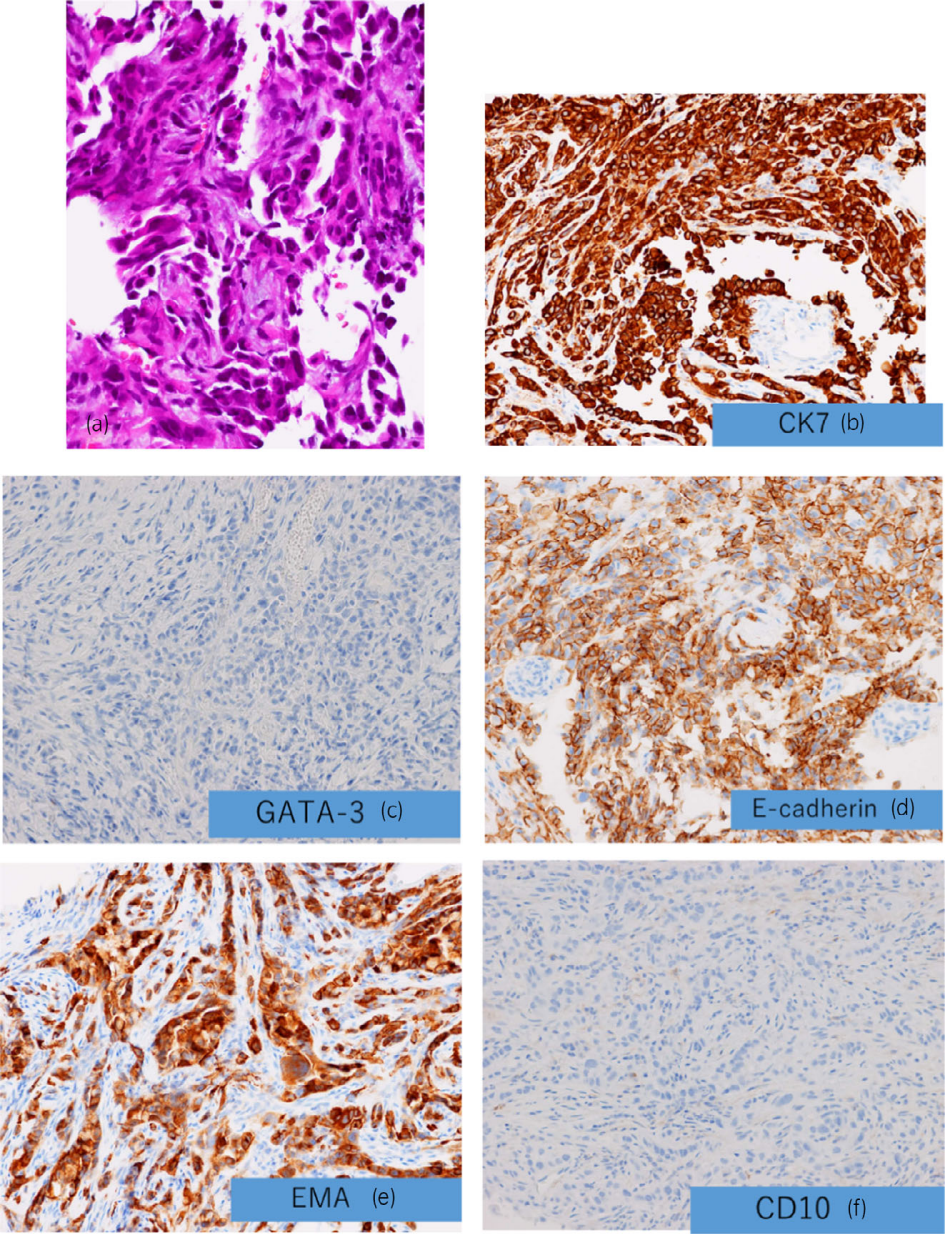
Pathology results. (a–f) Pathological results show atypical cell growth in cords and alveoli within the fibrous connective tissue with partial tube formation. Tumor cells are highly polymorphic (a). CK7 was positive (b). GATA‐3 was negative (c), indicating that urothelial carcinoma was unlikely. E‐cadherin (d), EMA (e), and CD10 (f) were positive, indicating a high probability of CDC.

The patient was a 69‐year‐old male with chief complaints of right‐sided chest pain and intermittent hematuria. An intrahepatic mass was suspected based on ultrasonography, and the patient was referred to the internal medicine department of our hospital. Based on contrast‐enhanced CT, the patient was diagnosed with renal cell carcinoma with a right‐sided thoracic wall and bilateral adrenal metastases (Fig. 1). The patient underwent a right‐sided thoracic wall metastasis biopsy and was diagnosed with CDC (Fig. 2) and referred to our department. Upon admission, the patient had a frail appearance and exhibited a palpable, hard mass on the right side of the chest. We diagnosed CDC stage cT2N1M1 which was categorized according to the International Metastatic Renal‐Cell Carcinoma Database Consortium prognostic risk categories as “poor risk”.

### Clinical course

**Fig. 3:**
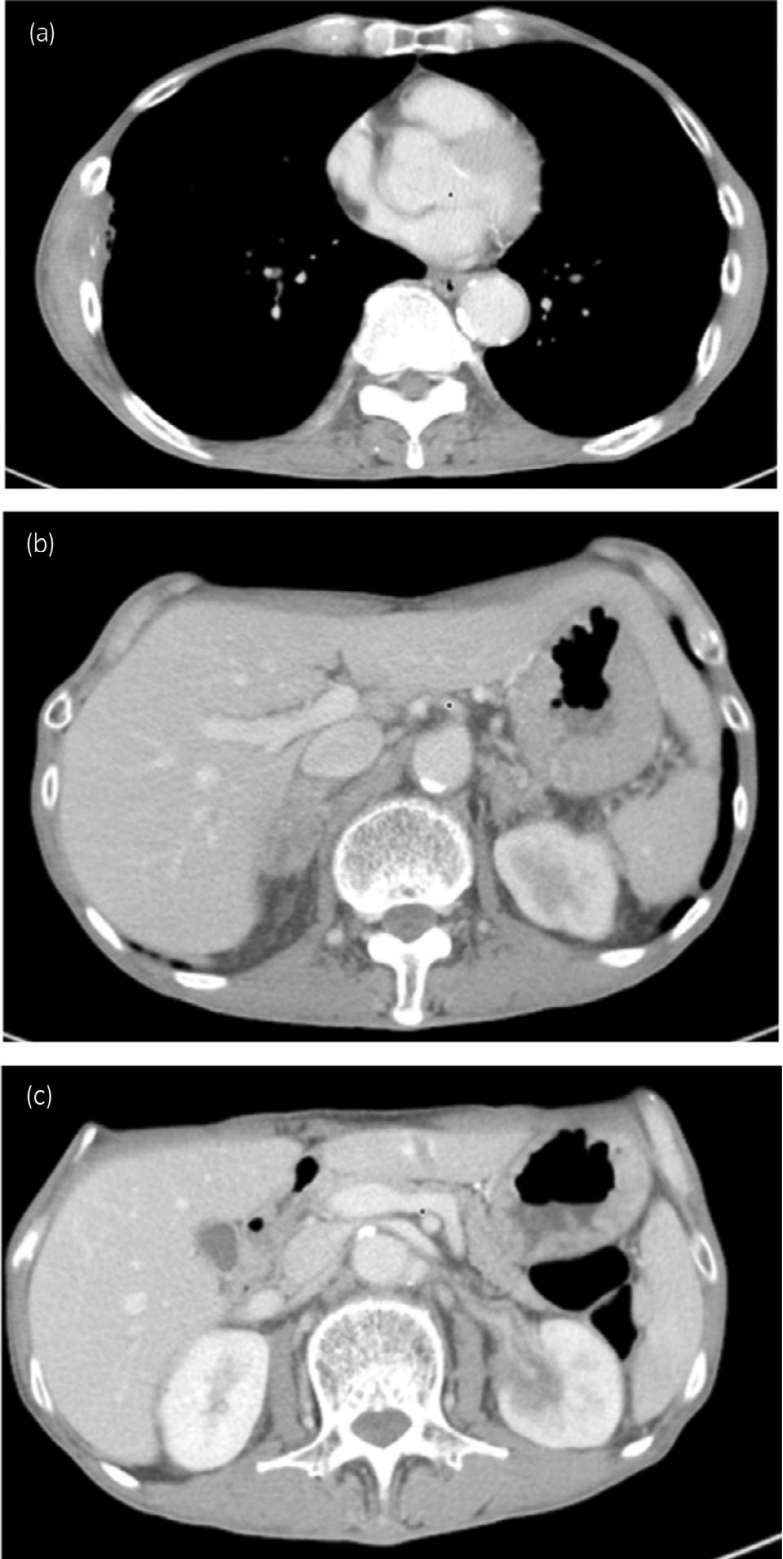
CT image 4 months after treatment. All tumor shrinkage was observed in the right thoracic wall (a), both adrenal glands (b), and the left kidney(c). We evaluated PR using RECIST 1.1.

**Fig. 4:**
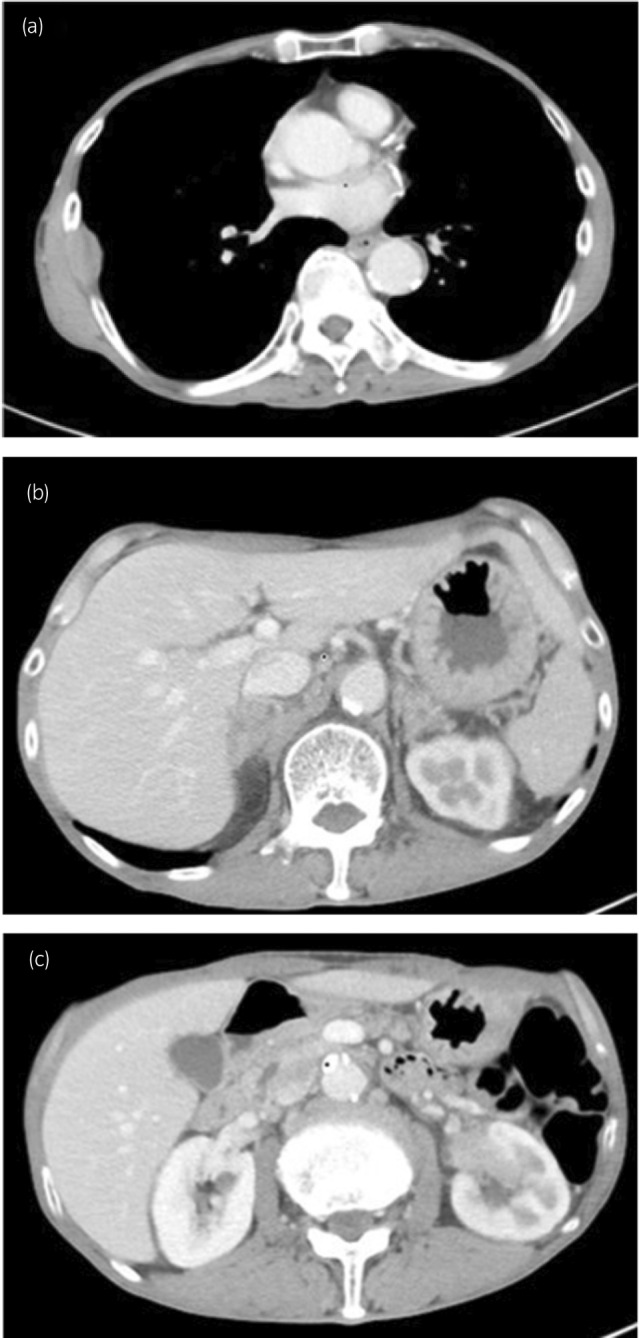
CT image on 7 months after treatment. The right thoracic wall metastasis (a) and the left kidney tumor (c) were enlarged. Both adrenal metastasis (b) remained unchanged in size. We evaluated PD using RECIST 1.1.

A combination of pembrolizumab plus axitinib was initiated. Axitinib was administered at 10 mg/day. Other than slight hoarseness, no obvious irAEs occurred. Oxycontin (10 mg/day) was administered for the right thoracic wall pain. Tumor shrinkage was observed 2 months after treatment initiation. In addition, the right chest pain decreased. Oxycontin was discontinued. The greatest tumor shrinkage was observed 4 months after treatment initiation, during which we evaluated PR (Fig. 3). Although shrinkage was maintained for several months, tumor growth was observed and evaluated PD at 7 months after treatment initiation, which we evaluated PD (Fig. 4). Forty milligrams of cabozantinib was selected as the second‐line therapy. However, 1 month after initial administration, the patient experienced left upper extremity pain and difficulty in lifting. The patient was diagnosed with a pathological fracture due to left humeral metastasis. We discontinued cabozantinib, and humeral fixation was performed by an orthopedic surgeon. The CT was reviewed retrospectively, and the humeral metastasis was also visible during the previous treatment. Therefore, we planned to continue the cabozantinib without considering that this treatment may have been ineffective. Two weeks after humeral fixation, engraftment was confirmed, and cabozantinib was resumed at the same dose. One month after resumption (9 months after the start of treatment), imaging evaluation revealed no increase or decrease in tumor size. The treatment was continued at the same dose. However, the patient began to complain of fatigue. The cabozantinib dose was tapered to 20 mg every other day. Imaging performed 13 months after the start of treatment revealed tumor enlargement. Due to the magnitude of the patient weight loss, fatigue, and frailty, we concluded that proceeding with cabozantinib and subsequent treatments would be difficult for the patient. After a total of 14 months of treatment, we consulted with the patient and his family and decided to transition to optimal supportive care. The patient died 1 month later, 15 months after the initiation of treatment.

## Discussion

CDC is a rare pathological form of renal cancer that accounts for 1–2% of all renal cancers. Most cases are diagnosed when radical resection is not possible. The prognosis is poor, with a survival of approximately 10 months.^1^ There is no established standard treatment; however, platinum‐based chemotherapy, included in the treatment guidelines, has been used in many previous cases.^2^, ^3^, ^4^ In the present case, frailty was severe at the time of consultation, and it was determined that the patient could not tolerate chemotherapy. Therefore, alternative treatments were considered. Some reports have shown that tyrosine kinase inhibitors can be effective for advanced CDC. However, the longest prognosis when using sunitinib was 12 months.^5^

In addition, one study reported a complete response in patients with advanced CDC with ipilimumab plus nivolumab treatment after nephrectomy,^6^ and a second report showed a therapeutic effect with nivolumab.^7^ For clear‐cell renal cell carcinoma, the KeyNote 426 study clearly showed that pembrolizumab plus axitinib provided an initial therapeutic effect and relieved symptoms.^8^

Based on the above findings, we determined that pembrolizumab plus axitinib would be more tolerable than chemotherapy by focusing on the avoidance of irAEs. Treatment was initiated after providing sufficient explanation and consent to the patient and family. The right‐sided chest pain improved without major irAEs, and a tumor reduction effect was observed for approximately 7 months.

In PD‐L1‐positive cases, ICI combination therapy has been suggested as a treatment to extend PFS,^9^ and the JAVELIN Renal 101 study reported that PFS was favorable in PD‐L1 positive groups.^10^ PD‐L1 positivity in patients also contributed to the selection of ICI combination therapy.

However, another case report by the CDC reported that pembrolizumab plus axitinib had an initial therapeutic effect in patients without PD‐L1 expression.^11^ Therefore, any significant association between PD‐L1 expression and initial therapeutic effect remains unclear.

After tumor growth was observed with pembrolizumab plus axitinib in the current case, we investigated the use of chemotherapy or molecular‐targeted drugs as second‐line therapies. Although the patient gained weight with the first‐line treatment, we were hesitant to choose chemotherapy because of continued frailty.

Cabozantinib has been reported as beneficial even after second‐line treatment for non‐clear cell cancers, including CDC^11^; one study showed that cabozantinib was effective in 35% of patients with CDC.^12^ Considering this, we chose cabozantinib as the second‐line treatment.

In the current case, anticancer effects were observed with cabozantinib at a dose of 40 mg. However, the patient's intolerance made continuing oral administration difficult, and the tumor size increased.

The patient was willing to undergo further treatment, and the next course of treatment was considered. However, due to exacerbation of frailty and a decline in performance status, we determined that tolerating further treatment was not possible. Cancer treatment was discontinued, and the patient was given optimal supportive care.

Had the patient tolerated the treatment, we may have considered sorafenib, everolimus, and other molecular‐targeted therapies that have been reported as effective in CDC as a supplemental treatment to platinum‐based chemotherapy.^13^

In the future, we hope that other case reports and clinical trials of effective treatments for metastatic CDC will be published and that treatment guidelines will be established.

## Conclusion

Our experience with this case showed that pembrolizumab plus axitinib may be a therapeutic option for symptomatic metastatic CDC.

## Author contributions

Yuichiro Atagi: Conceptualization. Kei Daizumoto: Writing – review and editing. Shinsuke Mohri: Supervision. Kai Somiya: Supervision. Daisuke Seto: Supervision. Shigeo Nakanishi: Supervision. Yutaka Yanagihara: Supervision. Iku Ninomiya: Supervision. Kenjiro Okamoto: Supervision. Sadamu Yamashi: Supervision.

## Conflict of interest

The authors declare no conflict of interest.

## Approval of the research protocol by an Institutional Reviewer Board

This research was approved by the hospital Institutional Reviewer Board.

## Informed consent

Informed consent was obtained from the patient and family.

## Registry and the Registration No. of the study/trial

Registration No. of the study/trial was 研04‐05 at Ehime Prefectural Central Hospital. This report conforms to the provisions of the Declaration of Helsinki (as revised in Fortaleza, Brazil, October 2013).

## Data Availability

Data from this case report will be available from the corresponding author upon reasonable request.
